# Non-aggregated Aβ25-35 Upregulates Primary Astrocyte Proliferation *In Vitro*

**DOI:** 10.3389/fncel.2017.00301

**Published:** 2017-09-26

**Authors:** Elise C. Ohki, Thomas J. Langan, Kyla R. Rodgers, Richard C. Chou

**Affiliations:** ^1^Department of Interdisciplinary Natural Sciences, Roswell Park Cancer Institute, State University of New York at Buffalo, Buffalo, NY, United States; ^2^Departments of Neurology, Pediatrics, and Physiology and Biophysics, Jacobs School of Medicine and Biomedical Sciences, State University of New York at Buffalo, Buffalo, NY, United States; ^3^Hunter James Kelly Research Institute, New York State Center of Excellence Bioinformatics & Life Sciences, Buffalo, NY, United States; ^4^Department of Medicine, Geisel School of Medicine at Dartmouth, Lebanon, NH, United States; ^5^Section of Rheumatology, Department of Medicine, Dartmouth-Hitchcock Medical Center, Lebanon, NH, United States

**Keywords:** astrocyte, amyloid beta, cell cycle, primary cultures, Alzheimer’s disease

## Abstract

Amyloid beta (Aβ) is a peptide cleaved from amyloid precursor protein that contributes to the formation of senile plaques in Alzheimer’s disease (AD). The relationship between Aβ and astrocyte proliferation in AD remains controversial. Despite pathological findings of increased astrocytic mitosis in AD brains, *in vitro* studies show an inhibitory effect of Aβ on astrocyte proliferation. In this study, we determined the effect of an active fragment of Aβ (Aβ_25-35_) on the cell cycle progression of primary rat astrocytes. We found that Aβ_25-35_ (0.3–1.0 μg/ml) enhanced astrocyte proliferation *in vitro* in a time- and concentration-dependent manner. Increased DNA synthesis by Aβ_25-35_ was observed during the S phase of the astrocyte cell cycle, as indicated by proliferation kinetics and bromodeoxyuridine immunocytochemical staining. Aggregation of Aβ_25-35_ abolished the upregulatory effect of Aβ on astrocyte proliferation. Further examination indicated that Aβ_25-35_ affected astrocyte proliferation during early or mid-G_1_ phase but had no effect on DNA synthesis at the peak of S phase. These results provide insight into the relationship between Aβ_25-35_ and astrocyte cell cycling in AD.

## Introduction

Alzheimer’s disease (AD), the most common form of neurodegenerative disease (Alzheimer’s Association, 2015), is characterized by two pathological hallmarks: senile plaques composed of amyloid beta (Aβ) and neurofibrillary tangles made up of hyperphosphorylated tau (Scheltens et al., 2016). Deposition of Aβ fibrils is an early finding in AD brains (Roth et al., 1966; Pike et al., 1995a; Rama Rao and Kielian, 2015). Aβ is a peptide that is proteolytically cleaved from transmembrane amyloid precursor protein (APP) by alpha, beta, or gamma secretases and can vary in length (LaFerla et al., 2007; Chow et al., 2010). Mutations in either APP or presenilins, which are the catalytic subunits of the gamma secretase complex, increase the risk for AD, possibly by elevating the production of abnormal APP cleavage products that are more prone to forming pathogenic fibrils (Iwatsubo et al., 1994, 1995; Gu and Guo, 2013). Several different Aβ fragments alter the functions of neurons and glia, including Aβ_25-35_ (Iversen et al., 1995; Millucci et al., 2010; Abeti et al., 2011; Dal Pra et al., 2011), Aβ_1-42_ (Iversen et al., 1995; Dal Pra et al., 2011; Blennow et al., 2015), Aβ_22-35_ (Takadera et al., 1993; Iversen et al., 1995), and Aβ_12-28_ (Iversen et al., 1995; Giulian et al., 1996). Each of these species has different propensities for forming aggregates and senile plaques (Kummer and Heneka, 2014). Among these Aβ_25-35_ was also shown to induce the production of Aβ_1-42_ in cultured human astrocytes (Dal Pra et al., 2011). Although Aβ is believed to be critical for the development of AD, the biology of different peptide species and their aggregates, as well as the mechanisms by which they drive pathogenesis remain largely unknown (De Strooper and Karran, 2016).

Interestingly, amyloid deposits and senile plaques are surrounded by reactive astrocytes (Pike et al., 1995a; Rama Rao and Kielian, 2015), a phenomenon noted by Alzheimer himself in his initial pathological analysis and recently confirmed by others (Alzheimer et al., 1995; Santillo et al., 2011). Although remaining in a state of quiescence under normal circumstances, mammalian astrocytes play critical roles in the biochemical and physiological functions of the central nervous system (Langan, 1993), including shaping synaptic transmission (Murphy-Royal et al., 2015), regulating cerebral blood flow (Zonta et al., 2003), and maintaining proper levels of extracellular ions and neurotransmitters (Simard and Nedergaard, 2004; Olsen et al., 2015). However, in AD and other pathological conditions, astrocytes undergo metabolic and phenotypic transformations, re-entering the cell cycle and actively proliferating, a state known as reactive astrogliosis (Amaducci et al., 1981; Pekny et al., 2016; Rodriguez-Arellano et al., 2016). Reactivation of astrocytes is thought to be a crucial early step in the pathogenesis of AD, and prevention of astrogliosis has been proposed as a potential therapeutic strategy (Pekny et al., 2014, 2016; Scholl et al., 2015; Rodriguez-Arellano et al., 2016). The mechanisms underlying cell-cycle re-entry in astrocytes and the relationship between different Aβ species, aggregation states, and reactive astrogliosis remain a matter of intense debate, and further investigation is necessary before novel therapeutic strategies can be proposed (Jordan-Sciutto et al., 2002; Gartner et al., 2003; Yang et al., 2006).

The goal of the present study was to gain a better understanding of the functional relationship between Aβ and proliferation of astrocytes. We hypothesized that soluble Aβ, but not aggregated (agAβ), promotes astrocyte reactivation regardless of cell-cycle phase. To test this hypothesis, we treated serum-deprived and non-serum-deprived rat astrocytes with Aβ_25-35_ and measured their rates of proliferation after varying lengths of time *in vitro*. We further investigated if aggregation of soluble Aβ_25-35_ alters its effects on astrocytes. Our results provide insight into the functional roles of Aβ_25-35_, as well as results of its aggregation, in reactive astrocytosis.

## Materials and Methods

### Primary Astrocyte Culture

All experimental procedures were approved by the Institutional Animal Care and Use Committee (IACUC) at the SUNY at Buffalo, and all experiments were carried out in accordance with the approved procedures. Newborn pups were purchased from Harlan Sprague-Dawley and housed briefly in the Animal Research Center, SUNY at Buffalo. The cerebral cortices of newborn (<48 h) Sprague-Dawley rat pups (Harlan Sprague Dawley, Indianapolis, IN, United States) were harvested and mechanically dissociated. Astrocytes were isolated by filtration through nylon mesh with 20 μm pore size to remove tissue debris (Langan and Slater, 1992; Langan and Chou, 2011). Primary astrocyte cultures were maintained in 10% fetal bovine serum (Hyclone, Logan, UT, United States) and 1% penicillin–streptomycin (v/v; Sigma, St. Louis, MO, United States) in Dulbecco’s modified Eagle’s medium (DMEM) (Gibco/Life Technologies, Inc., Grand Island, NY, United States) at 37°C in a CO_2_ incubator (5% CO_2_/95% humidified air) until *in vitro* studies. A previous study shows that the initial astrocyte cultures generated by this method are of ≥95% purity (Langan and Chou, 2011; Langan et al., 2017).

### Astrocyte Subculture and *In Vitro* Stimulation

#### Non-serum-Deprivation Experiments

Primary astrocytes were harvested by trypsinization and passaged into 6-well plates at a concentration of 1 × 10^4^ cells/cm^2^ in 10% bovine calf serum (BCS; Hyclone)/DMEM (v/v). After an initial 2-h incubation at 37°C in 5% CO_2_/95% humidified air, the medium was removed, and cell cultures were washed once with phosphate-buffered saline (PBS, pH 7.4) to remove cell debris and non-adherent cells; 3 ml of 10% BCS/DMEM was then added to adherent cells. Astrocytes were allowed to proliferate at 37°C in 5% CO_2_/95% humidified air for 48 h. At the end of incubation, the supernatant was replaced with 2 ml fresh 10% BCS/DMEM in the presence of varying concentrations of agAβ_25-35_ (in PBS, pH 7.4), freshly prepared non-agAβ_25-35_ (in PBS, pH 7.4), or control peptide (CP; Cal-Biochem, La Jolla, CA, United States) (in PBS, pH 7.4), which was the Aβ fragment of interest with the amino acids in reverse order (i.e., Aβ_35-25_). This step represented the start of the experiment (i.e., *T*_0_).

#### Serum-Deprivation Experiments

Astrocytes were passaged into 6-well plates at a concentration of 1 × 10^4^ cells/cm^2^ in 10% BCS/DMEM. Cells were then allowed to grow to 30–50% confluence in 10% BCS/DMEM at 37°C in 5% CO_2_/95% humidified air. At the end of incubation, the medium was removed, and cells were rinsed with PBS (pH 7.4). Cells were then overlaid with 3 ml 0.1% BCS/DMEM and incubated at 37°C in 5% CO_2_/95% humidified air for 48 h, such that by the end of the incubation period, 85–90% of cells entered cell-cycle arrest (Langan and Slater, 1991; Chou and Langan, 2003; Langan and Chou, 2011; Langan et al., 2017). At the end of the serum-deprivation process, the medium was replaced with 2 ml 10% BCS/DMEM. This serum up-shift allowed astrocytes to re-enter the cell cycle in a first-order manner and represented the start of the cell-cycle entry experiment (i.e., *T*_0_) (Langan and Volpe, 1987; Kniss and Burry, 1988; Chou and Langan, 2003; Langan and Chou, 2011; Langan et al., 2017). Varying concentrations of agAβ_25-35_, non-aggregated (freshly prepared) Aβ_25-35_, or CP were added to the cultures concurrently with the medium, allowing astrocytes to re-enter the cell cycle under defined experimental conditions.

### Aβ_25-35_ and Its Aggregation

The Aβ_25-35_ peptide is a truncated product of Aβ that was shown to be physiologically active, and was widely published in articles using rat models of AD (Soto et al., 1998; Arif et al., 2009; Fedotova et al., 2016; Nell et al., 2016; Soultanov et al., 2016). Others have shown that the effects of Aβ_25-35_ on different cellular functions in both astrocytes and neurons are similar to those of Aβ_1-42_ (Chiarini et al., 2010; Kaminsky et al., 2010); additionally, although its physiologic role remains unclear, histopathological studies have shown that Aβ_25-35_ is present in senile plaques of Alzheimer’s brains (Kubo et al., 2003). For these reasons, Aβ_25-35_ is the focus of the present studies using rat astrocytes. Quantitative analysis of Aβ_25-35_ aggregation by sedimentation assay was performed according to an established procedure (Pike et al., 1994, 1995b). In brief, the lysine residues in the non-agAβ_25-35_ fragment react with the fluorescent marker fluorescamine (FLCN; Molecular Probes, Inc., Eugene, OR, United States). Aggregation of the Aβ_25-35_ fragments is measured by a decrease in the number of lysine residues available to bind to FLCN, resulting in a reduction in fluorescence intensity proportional to the extent of aggregation. Different concentrations of Aβ_25-35_ and CP were solubilized in 1 ml PBS (pH 7.4) at room temperature (RT) for 2 or 48 h. Aliquots of the FLCN solution in acetonitrile were added to each set of samples to achieve a 1:1 mol/ml ratio of peptide to FLCN. Following the addition of FLCN, half of the samples were measured for FLCN fluorescence without centrifugation, and half were centrifuged at 100,000 × *g* for 1 h, and the supernatant was used to measure FLCN fluorescence (Pike et al., 1995b). Fluorescence was measured at 478 nm by exciting the peptide at 383 nm with a fluorescence (LS-5) spectrophotometer (Perkin-Elmer, Norwalk, CT, United States) (De Bernardo et al., 1974; Alavi et al., 2013).

### Cell Proliferation Assays

#### ^3^H-Thymidine Incorporation Assay

The incorporation of tritiated [methyl-^3^H]-thymidine into primary astrocytes was used to quantify cell proliferation according to established procedures (Langan and Volpe, 1987; Langan and Slater, 1992; Chou and Langan, 2003; Langan and Chou, 2011; Langan et al., 2017). Radio-labeled [methyl-^3^H]-thymidine (25 Ci/mmol; Amersham, Arlington Heights, IL, United States) was added to each well 1 h prior to the termination of the experiment at a final activity of 1.0 μCi/ml (37°C, 5% CO_2_/95% humidified air). At the end of the incubation, cultures were washed with 2 ml Tris–EDTA buffer (pH 7.4) twice to remove any excess ^3^H-thymidine. DNA and total cellular protein were extracted using the trichloroacetic acid precipitation method (Langan and Volpe, 1987; Langan and Slater, 1992). Cell proliferation was measured as the incorporation of radioactivity per microgram of protein present in the acid-precipitated portion (cpm/μg protein). Tritium was quantified in the samples with a beta counter (LKB Wallac, Gaithersburg, MD, United States) for 10 min using an Ecoscint-A liquid scintillation cocktail (National Diagnostics, Manville, NJ, United States), and the total cellular protein in the samples was determined by Bradford assay (BioRad, Hercules, CA, United states) using a microplate reader (model 3550-UV; BioRad, Hercules, CA, United states) at a wavelength of 595 nm.

#### BrdU Incorporation Assay

The number of cells undergoing active DNA synthesis was quantified by immunocytochemical staining for bromodeoxyuridine (BrdU) (Sigma) (Yong et al., 1988; Langan and Slater, 1991). Primary astrocyte cultures were passaged onto 4.9 mm^2^ glass cover slips coated with poly-lysine (Sigma) in 12-well plates, grown to 30–50% confluency, and rendered into cell cycle arrest by serum deprivation as described above. Serum-deprived astrocytes were then allowed to re-enter the cell cycle under experimental conditions. To identify cells undergoing active DNA synthesis, BrdU was added to cell cultures at a final concentration of 15 μM for a period of 2.5 h prior to the termination of experiments. At the end of incubation, the cover slips were removed and rinsed with PBS (pH 7.4) followed by fixation with 4% paraformaldehyde (PFA; Sigma) in PBS (pH 7.4) for 10 min at RT. After fixation, the 4% PFA was aspirated, and cells were washed twice with PBS (pH 7.4). Fixed cells were then treated with 2 M HCl (10 min, RT) to permeabilize the nuclear membrane. The HCl was removed, and cells were again washed twice with PBS (pH 7.4) followed by incubation with 0.1 M NaB_4_O_7_ to neutralize the HCl (10 min, RT). After acid neutralization, the NaB_4_O_7_ was removed, and cells were washed three times with PBS (pH 7.4). Next, cells were blocked with 5% bovine serum albumin in PBS (20 min, RT). Monoclonal antibody for BrdU (Sigma) was added at a dilution of 1:200 for 1 h at RT followed by the addition of goat anti-rat rhodamine-labeled secondary antibody (Sigma) at a dilution of 1:200 for 30 min. The percent of BrdU+ nuclei was calculated based on the cell count in each of the randomly selected high powered fields using a phase-fluorescent microscope (Langan and Slater, 1991; Li et al., 1996).

### Cell Viability

In parallel to the cell proliferation assays, an identical set of cells was subcultured and treated as described above for each set of experiments. At the end of each time point, cells were harvested by trypsinization, centrifuged, and resuspended in PBS. A 0.4% trypan blue solution (Sigma) was added to the cell suspension in a 1:1 v/v ratio. A manual cell count was performed using a hemocytometer.

### Statistical Analysis

All experiments were repeated at least three times, and all conditions were performed in triplicate. Data were expressed as mean ± standard error of the mean (SEM) and analyzed using Student’s *t*-tests for unpaired comparisons and one-way ANOVA for multiple group comparisons. Statistical significance was defined as *p* < 0.05.

## Results

### Aβ_25-35_ Upregulates Proliferation of Non-serum-Deprived Astrocytes in a Time- and Concentration-Dependent Manner

**FIGURE 1:**
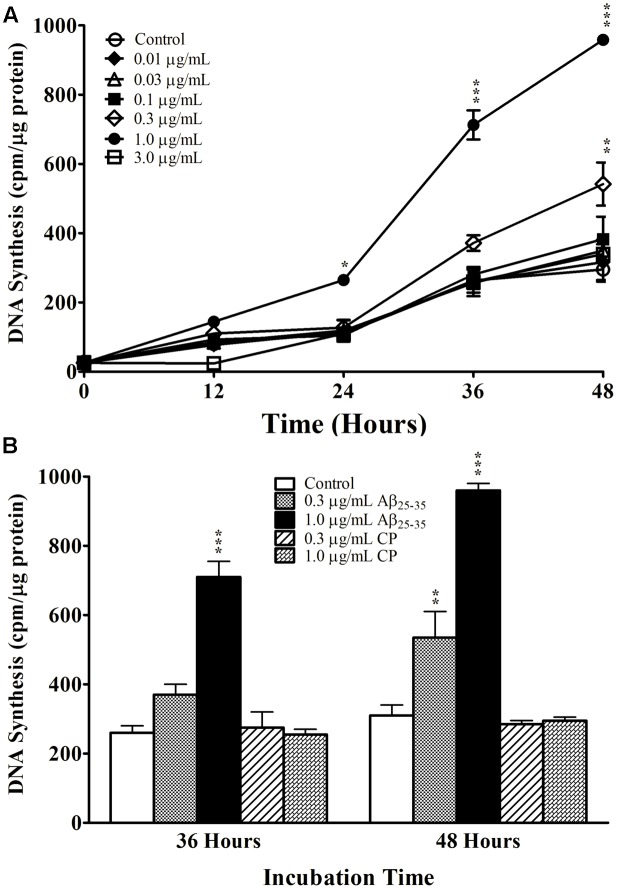
The effect and specificity of Aβ_25-35_ on proliferation of non-serum-deprived primary astrocytes. **(A)** Subcultured astrocytes were incubated with graded concentrations of freshly prepared and solubilized Aβ_25-35_ or control. ^3^H-thymidine was added to cultures 1 h prior to termination. Cultures were terminated every 12 h and processed for ^3^H-thymidine uptake to measure rate of DNA synthesis. **(B)** Subcultured astrocytes were incubated with freshly prepared Aβ_25-35_ or CP at various concentrations for 36 or 48 h. Rate of DNA synthesis was measured using ^3^H-thymidine incorporation assay. ^∗^*p* < 0.05; ^∗∗^*p* < 0.02; ^∗∗∗^*p* < 0.001 (results were average values of three representative experiments).

We first examined the effect of Aβ_25-35_ on astrocyte proliferation by measuring DNA synthesis of non-serum-deprived primary astrocytes treated with freshly prepared and graded concentrations of Aβ_25-35_ (0.01–3.0 μg/ml). Astrocytes proliferated in an exponential-like manner over a 48-h period, with a lag phase in the initial 24 h, a log phase in the next 12 h (24–36 h), and a slowed rate of proliferation in the last 12 h tested (36–48 h). We found that Aβ_25-35_ increased the rate of DNA synthesis in primary astrocytes in a time- and concentration-dependent manner (**Figure 1A**). At a concentration of 0.3 μg/ml, Aβ_25-35_ only caused a significant increase in DNA synthesis after 48 h of incubation compared with control. The greatest effect on astrocyte proliferation was obtained using a concentration of 1.0 μg/ml Aβ_25-35_, which caused significant increases in DNA synthesis after 24, 36, and 48 h of incubation. A further increase in the concentration of Aβ_25-35_ to 3.0 μg/ml did not upregulate the rate of astrocyte proliferation at any time point, which therefore followed a classical concentration-effect response (**Figure 1A**). Based on these findings, we utilized Aβ_25-35_ at a concentration of 1.0 μg/ml in subsequent experiments.

We next examined the specificity of the response to Aβ_25-35_ by treating primary astrocytes with either Aβ_25-35_ or CP consisting of the reversed amino acid sequence (i.e., Aβ_35-25_) at varying concentrations for 36 or 48 h. CP did not upregulate DNA synthesis in primary astrocyte cultures compared with control at either time point or either concentration tested (**Figure 1B**), supporting the notion that the increased proliferation of astrocytes in response to Aβ_25-35_ depends on its amino acid sequence.

### Aβ_25-35_ Upregulates DNA Synthesis During S Phase in Serum-Deprived Primary Astrocytes

**FIGURE 2:**
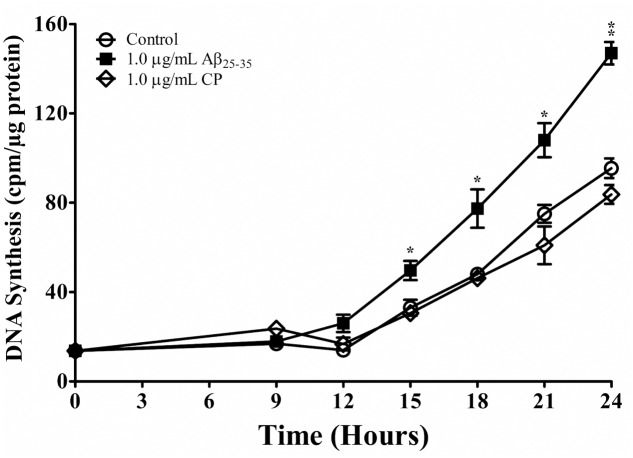
The effect of Aβ_25-35_ on proliferation of serum-deprived primary astrocytes. Newborn astrocytes were rendered into G_0_ phase by serum deprivation and then allowed to re-enter the cell cycle via serum up-shift in the presence of freshly prepared Aβ_25-35_ (1.0 μg/ml) or CP (1.0 μg/ml). ^3^H-thymidine incorporation assay was performed to measure rate of DNA synthesis. ^∗^*p* < 0.05; ^∗∗^*p* < 0.02; ^∗∗∗^*p* < 0.001 (results were average values of three representative experiments).

We next addressed whether Aβ_25-35_ affects cell-cycle kinetics of astrocytes *in vitro*. Astrocytes were first subcultured in serum-deprived medium that rendered cell-cycle arrest. Astrocytes were then exposed to serum up-shift (Langan and Chou, 2011; Langan et al., 2017), resulting in re-entry into the cell cycle with highly replicable kinetics (**Figure 2**). Consistent with previous studies (Langan and Slater, 1992; Langan, 1993), following serum up-shift, astrocytes remained in G_1_ phase for 12 h and then entered the S phase for the next 12 h, as shown by a first-order increase in DNA synthesis (**Figure 2**). When astrocytes re-entered the cell cycle in the presence of Aβ_25-35_ (1 μg/ml), the rate of proliferation was significantly upregulated compared with CP-treated astrocytes (**Figure 2**). Aβ_25-35_ did not impact the length of G_1_ or increase the rate of proliferation of astrocytes during this time, suggesting that the effect of Aβ_25-35_ on astrocytes is restricted to S phase. Additionally, altered proliferation in serum-deprived astrocytes was specifically mediated by Aβ_25-35_, as CP did not significantly impact DNA synthesis compared with control (**Figure 2**).

**FIGURE 3:**
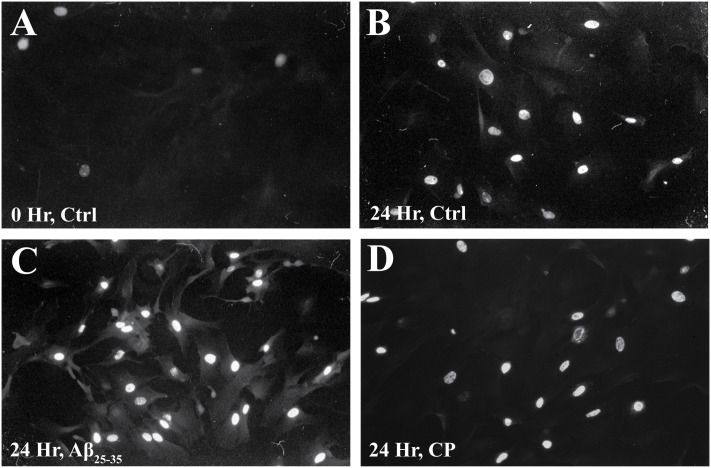
The effect of Aβ_25-35_ on BrdU incorporation in serum-deprived primary astrocytes. Newborn astrocytes were serum deprived for 48 h, resulting in cell cycle arrest, as demonstrated by low BrdU+ cell count (**A**; 18.38 ± 0.02%, *n* = 9, total cell count = 102). Following serum deprivation, cells were stimulated to re-enter the cell cycle in the presence of either freshly prepared Aβ_25-35_ (1.0 μg/ml) or CP (1.0 μg/ml). BrdU was added to the cultures 2.5 h prior to termination. After 24 h of incubation, samples were processed for immunocytochemical staining of BrdU. There was no significant difference in the number of BrdU+ cells between the control (**B**; 65.42 ± 0.02%, *n* = 11, total cell count = 104) and CP-treated astrocytes (**D**; 62.44 ± 0.02%, *n* = 11, total cell count = 100). Significantly more Aβ_25-35_-treated astrocytes were BrdU+ (**C**; 87.35 ± 0.02%; *n* = 11, total cell count = 102, *p* < 0.001) compared to controls. Images were taken with fluorescence microscopy.

We confirmed these findings using immunocytochemical staining for BrdU (**Figure 3** and **Table 1**) and quantifying the percent of BrdU+ cells at *T*_0_ and *T*_24_ in the presence or absence of Aβ_25-35_ (1 μg/ml). Following 48 h of serum deprivation (i.e., *T*_0_), more than 81% of cells were in a state of quiescence (**Figure 3** and **Table 1**). At *T*_24_, the peak of S phase, about 65% of the cells were BrdU+, indicating that they had re-entered the cell cycle and undergone active proliferation (**Figure 3** and **Table 1**). In the presence of Aβ_25-35_, significantly more astrocytes re-entered the cell cycle, as indicated by BrdU+ staining (**Figure 3** and **Table 1**). This increase in the percentage of BrdU+ cells was specific to Aβ_25-35_ treatment, as there was no significant change in the percentage of BrdU+ cells after 24 h of incubation with CP (**Figure 3** and **Table 1**) compared to control.

**Table 1:** Percent of BrdU+ astrocytes in response to various treatments for 24 h.

Treatment	Time point (hour)	Mean ± SEM (%)
Control	0	18.38 ± 0.02
	24	65.42 ± 0.02
Aβ_25-35_	24	87.35 ± 0.02^∗^
CP	24	62.44 ± 0.02


*^∗^*p* < 0.005.*

### Aggregation of Aβ_25-35_ Impairs Its Effect on Proliferation Rate in Non-serum-Deprived Astrocytes

**FIGURE 4:**
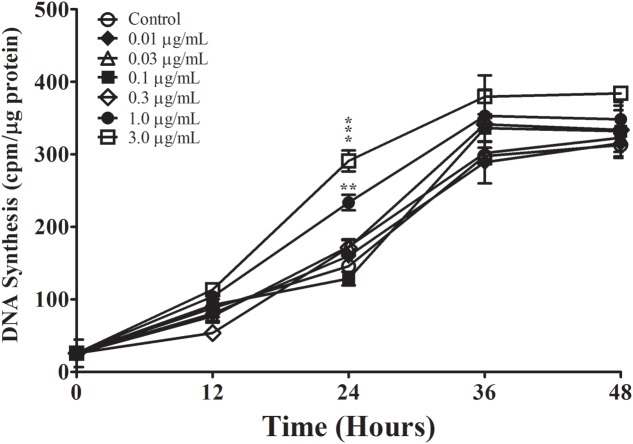
The effect of Aβ_25-35_ aggregation on the proliferation of non-serum-deprived primary astrocytes. Freshly prepared Aβ_25-35_ was allowed to aggregate in PBS (pH 7.4) for 48 h; aggregation was confirmed by FLCN incorporation assay (**Table 2**). Non-serum-deprived astrocytes were treated with varying concentrations of agAβ_25-35_ (0.01–3.0 μg/ml), and ^3^H-thymidine incorporation assay was performed to measure rate of DNA synthesis. ^∗^*p* < 0.05; ^∗∗^*p* < 0.02; ^∗∗∗^*p* < 0.001 (results were average values of three representative experiments).

In order to generate agAβ_25-35_, we allowed graded concentrations of Aβ_25-35_ to aggregate for 2 or 48 h; aggregation was confirmed by measuring FLCN incorporation. Aggregation was compared in two different manners: (1) time-dependent effect in the non-centrifugation portion, i.e., 2 vs. 48 h, as represented by the statistical symbol “^∗^” and (2) concentration-dependent effect in each time point, i.e., suspension vs. supernatant, as represented by the statistical symbol “^†^”. We observed a significant decrease in fluorescence after 48 h at all concentrations of Aβ_25-35_ (0.5–10.0 μg/ml) when compared with 2 h incubation (**Table 2**), both in the non-centrifuged solution and in the supernatant of the centrifuged solution. These data suggest that incubating the peptide at RT for 48 h results in the formation of aggregates, as evidenced by the decreased FLCN absorbance in the supernatants of samples that were solubilized for 48 h as compared to those that were solubilized for 2 h prior to centrifugation. Based on our findings, non-centrifuged solutions were used for downstream experiments in order to ensure that astrocytes were exposed to aggregates of varying sizes. To address the question of whether oligomerization of Aβ_25-35_ affects astrocyte proliferation, we treated non-serum-deprived primary astrocyte cultures for 12, 24, 36, or 48 h with graded concentrations of agAβ_25-35_ or non-agAβ_25-35_. In contrast to our earlier findings using Aβ_25-35_ (**Figure 1A**), agAβ_25-35_ only significantly increased the rate of proliferation of non-serum-deprived astrocytes compared with control after 24 h of stimulation at a concentration of 3.0 μg/ml (**Figure 4**).

**Table 2:** Effect of time and fraction on Aβ_25-35_ aggregation in solution^‡^.

[Aβ_25-35_] (μg/ml)	Centrifugation	2 h, mean fluorescence intensity ± SEM	48 h, mean fluorescence intensity ± SEM
0.5	-	3.20 ± 1.21	1.20 ± 0.18^∗^
	+	2.00 ± 0.77	0.9 ± 0.08^∗†^
1.0	-	11.40 ± 0.63	4.00 ± 0.31^∗∗∗∗^
	+	9.60 ± 2.44	3.60 ± 0.85^∗∗∗†††^
3.0	-	34.20 ± 1.76	22.70 ± 0.76^∗∗^
	+	21.5 ± 4.00	9.00 ± 0.76^∗∗††^
10.0	-	187.5 ± 5.46	99.20 ± 4.41^∗∗∗∗^
	+	136.50 ± 4.37	47.20 ± 2.73^∗∗∗∗††††^


*^‡^Fluorescence intensity is measured in arbitrary units.; ^∗†^*p* < 0.025; ^∗∗††^*p* < 0.001; ^∗∗∗†††^*p* < 0.005; ^∗∗∗∗††††^*p* < 0.0005.*

### Exposure to Aβ_25-35_ During G_1_ Phase Is Required to Stimulate Proliferation of Serum-Deprived Astrocytes

**FIGURE 5:**
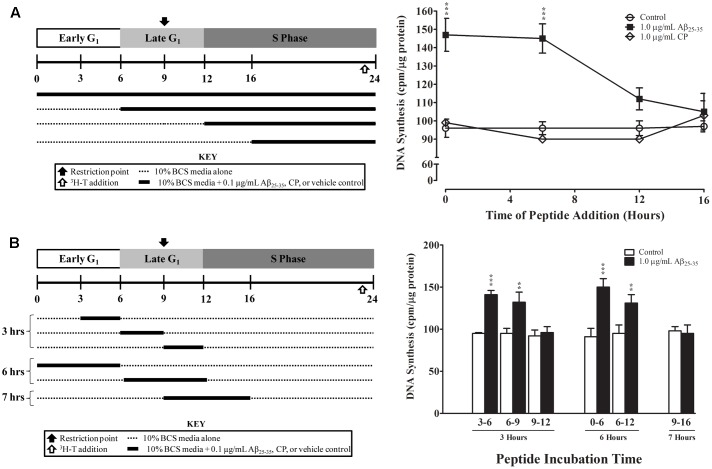
Temporal effects of Aβ_25-35_ on the proliferation of serum-deprived primary astrocytes. Newborn astrocytes were first serum-deprived and then re-entered into the cell cycle by serum up-shift. **(A)** Left, experimental approach; Right, Aβ_25-35_ (1.0 μg/ml) or CP (1.0 μg/ml) was added to the cultures at varying times after serum up-shift (0, 6, 12, or 16 h). **(B)** Left, experimental approach; Right, Aβ_25-35_ (1.0 μg/ml) was added to the cultures at different time points and then removed via medium changes after 3, 6, or 7 h of incubation. All cultures were terminated at *T*_24_, and ^3^H-thymidine incorporation assay was performed to measure rate of DNA synthesis. ^∗^*p* < 0.05; ^∗∗^*p* < 0.02; ^∗∗∗^*p* < 0.001 (results were average values of three representative experiments).

Finally, we examined whether the effect of Aβ_25-35_ on astrocyte proliferation is phase-specific. Aβ_25-35_ or CP (1.0 μg/ml) was added to serum-deprived astrocyte cultures at the time of serum up-shift or various times after serum up-shift (*T*_6_, *T*_12_, and *T*_16_). All cultures were terminated at the peak of the S phase (*T*_24_), and the rates of proliferation were determined using ^3^H-thymidine incorporation. Consistent with our earlier results, we found that adding Aβ_25-35_ at *T*_0_ significantly increased the rate of DNA synthesis at the peak of S phase (*T*_24_) compared with control or CP (*p* < 0.001; **Figure 5A**). When Aβ_25-35_ was added in the middle of G_1_ phase at *T*_6_, we observed a similar increase in the rate of DNA synthesis compared with CP or control (*p* < 0.02). However, when addition was delayed until the G_1_/S phase transition point (*T*_12_) or until the initiation of S phase, the rates of astrocyte proliferation were not significantly different among the Aβ_25-35_, CP, and control groups (**Figure 5A**), suggesting that the effect of Aβ_25-35_ on astrocyte proliferation is phase-specific, and initiated during the G_1_ phase and before G_1_/S phase transition point.

We further defined the critical period of Aβ_25-35_ exposure by using a “delayed addition and early removal” paradigm. In these experiments, we added Aβ_25-35_ to the cultures at different points of the cell cycle and then changed the medium to remove Aβ_25-35_ after varying exposure durations (3, 6, or 7 h); all cultures were terminated at the peak of the S phase (*T*_24_), and the rate of proliferation was determined using ^3^H-thymidine incorporation. We found that Aβ_25-35_ significantly increased the rate of astrocyte proliferation as compared to control only if it was added to the culture during early to mid-G_1_ phase (*T*_0_, *T*_3_, or *T*_6_; **Figure 5B**). When Aβ_25-35_ was added to the cultures in late G_1_ phase (*T*_9_), there was no significant difference in the rate of DNA synthesis compared with control (**Figure 5B**), regardless of whether the exposure duration was 3 (*T*_9_–*T*_12_) or 7 h (*T*_9_–*T*_16_). CP did not alter the rate of DNA synthesis during any of these exposure periods (data not shown). These results suggest that Aβ_25-35_ affects the proliferation of astrocytes during early-to-mid-G_1_ phase.

## Discussion

A definitive diagnosis of AD is based on histopathological evidence at autopsy or brain biopsy, including the presence of Aβ deposits, neuritic changes with formation of paired helical filaments and hyperphosphorylated tau, and reactive astrogliosis (Serrano-Pozo et al., 2011). Astrocytosis is widespread in the early phases of AD but declines as the disease progresses, indicating that gliosis may be an early pathogenic event (Pike et al., 1995a; Santillo et al., 2011; Rodriguez-Vieitez et al., 2015, 2016; Scholl et al., 2015). *In vitro* studies consistently demonstrate that Aβ is neurotoxic and consequently induces neuritic changes (Sendrowski et al., 2015; Sadleir et al., 2016). Although pathological studies show that Aβ-containing senile plaques are often surrounded by reactive astrocytes, the relationship between different Aβ species and functional changes in astrocytes is less clear. Here, we hypothesize that Aβ_25-35_ increases astrocyte proliferation, but that aggregation of Aβ_25-35_ inhibits this effect.

Interestingly, although aberrant expression of mitotic regulators, such as cyclin c and binding partner Cdk8 have been observed in the astrocytes of AD brains (Ueberham et al., 2003), *in vitro* studies demonstrate highly variable and contrasting effects of Aβ on astrocytes (Agostinho et al., 2010), including induction of apoptosis (Brera et al., 2000; Hou et al., 2011; Saha and Biswas, 2015) and oxidative stress (Brera et al., 2000), as well as changes in morphology (Salinero et al., 1997) and proliferation (Hernandez-Guillamon et al., 2009). Our results show that Aβ_25-35_ upregulates proliferation in both serum-deprived and non-serum-deprived astrocytes in time- and concentration-dependent manners as measured by ^3^H-thymidine incorporation assay and BrdU staining (**Figures 1**, **2**); importantly, Aβ_25-35_ did not induce cellular death in our experiments, as demonstrated by minimal cell death in trypan blue exclusion assay (<2%). We found that CP (Aβ_35-25_) did not impact astrocyte proliferation, confirming the bio-specific effect of Aβ_25-35_ on the rate of DNA synthesis in primary astrocytes depends on its amino acid sequence (**Figures 1**, **2**). Although increasing the concentration of Aβ_25-35_ upregulated the rate of proliferation over a certain range (i.e., 0.01–1.0 μg/ml), showing a clear concentration-effect of Aβ_25-35_ on DNA synthesis in astroctyes, further increase of the Aβ_25-35_ concentration to 3.0 μg/ml failed to enhance DNA synthesis (**Figure 1**). While our data demonstrate a narrow concentration-effect window, this phenomenon has been reported in other biological systems, including that seen in astrocytes. For example, it was shown that just a 1% change in the concentration of isoflurane had a dramatic effect on astrocyte calcium signaling in ferret visual cortex (Schummers et al., 2008). Similarly, it has been shown that mitochondrial function is extremely sensitive to a narrow range of Ca^2+^ ion concentrations (Schild et al., 2005), as is cellular survival in response to free zinc ions (Bozym et al., 2010). While the mechanisms underlying the concentration effects of each of these phenomena are currently unknown, one potential explanation of our data is that Aβ_25-35_ follows a classical concentration-effect response via specific receptors; however, further research is required in order to elucidate the molecular basis of this response (Salinero et al., 1997; Brera et al., 2000; Hou et al., 2011; Saha and Biswas, 2015). Our data showing narrow effective concentration range on astrocyte proliferation do suggest a plausible explanation for astrocyte toxicity in advanced AD. In fact, others have shown that Aβ_25-35_ is cytotoxic to astrocytes, those studies were carried out using concentrations 1000-fold higher than our current study; therefore, our results do not exclude the findings that high doses of Aβ_25-35_ can be cytotoxic to astrocytes *in vitro*.

Our findings also demonstrate that the effect of Aβ_25-35_ is cell cycle phase-specific. First, Aβ_25-35_ increases the rate of DNA synthesis in serum-deprived astrocytes during the S phase but not during G_1_ phase (**Figure 2**). Second, the timing of exposure to Aβ_25-35_ was critical. To upregulate astrocyte proliferation, Aβ_25-35_ needed to be added to astrocytes during early-to-mid-G_1_ phase. If Aβ_25-35_ was added any later than 6 h into G_1_, the rate of proliferation was not impacted (**Figure 5**). These findings offer novel insights into the effect of Aβ_25-35_ on astrocyte proliferation, and the mechanisms underlying the phase specificity are currently under investigation in our laboratory.

We showed that aggregation of Aβ_25-35_ impaired its upregulatory effect on astrocyte proliferation, as agAβ_25-35_ increased DNA synthesis relative to control only at a concentration of 3.0 μg/ml and only at *T*_24_ (i.e., 12 h into the S phase, **Figure 4**). Senile plaques, a hallmark feature of AD pathology, are made up of fibrillar or β-sheet aggregates of amyloid peptide; amyloid deposition has been shown to occur progressively over the course of the disease and has been proposed as a biomarker for disease staging (Jack et al., 2013). However, our results clearly show that aggregation of amyloid peptide limits its ability to stimulate astrocytes. As astrocyte activation precedes plaque formation in AD patients (Rodriguez-Vieitez et al., 2015), it is conceivable that initial Aβ deposition increases astrocyte proliferation and leads to reactive astrocytosis during the early stages of amyloid deposition, but that as senile plaques by amyloid aggregation are formed, the rate of astrocyte reactivation slows down.

## Conclusion

Our study is the first to show evidence of the upregulatory effect of Aβ_25-35_ on DNA synthesis during the S phase of the primary astrocyte cell cycle. Our results demonstrate that Aβ_25-35_ affected the cell cycle only when astrocytes were exposed during early-to-mid-G_1_ phase. Exposing primary astrocytes to Aβ_25-35_ 3 h prior to G_1_/S intersection (*T*_9_) failed to increase DNA synthesis in the S phase, irrespective of exposure duration. Therefore, these results suggest the temporal importance of Aβ exposure preceding the late G_1_ phase restriction point of the astrocyte cell cycle. The time-based specificity of the effect of Aβ_25-35_ on the astrocyte cell cycle thus points to the possibility that this peptide participates in the complex array of biochemical and molecular events that confer commitment to cell-cycle progression; further investigation is required to determine the exact mechanisms responsible. Additionally, while the present study focuses on the effect of Aβ_25-35_ on astrocytes, it would also be worthwhile to examine its impact on the cellular functions and rate of proliferation of other cells important for brain function, such as microglia, which have crucial roles in central nervous system immunity, and endothelial cells, which maintain the blood–brain barrier.

## Author Contributions

Experiments were conceived and designed by EO, TL, and RC. Experiments were performed by EO and RC. Data were analyzed by EO, KR, and RC. Reagents/materials/tools provided by TL. Manuscript was written and prepared by EO, KR, and RC. All authors reviewed and approved the final version of the manuscript.

## Conflict of Interest Statement

The authors declare that the research was conducted in the absence of any commercial or financial relationships that could be construed as a potential conflict of interest.
